# Effect of chemotherapy on passenger mutations in metastatic colorectal cancer

**DOI:** 10.1002/1878-0261.70154

**Published:** 2025-11-05

**Authors:** Marium T. Siddiqui, Matthew A. Cottam, Muhammad Bilal Mirza, Keeli B. Lewis, Kristen K. Ciombor, Mary Kay Washington, Kamran Idrees

**Affiliations:** ^1^ Department of Surgery, Division of Surgical Oncology and Endocrine Surgery Vanderbilt University Medical Center Nashville TN USA; ^2^ Division of Hematology and Oncology, Department of Internal Medicine Vanderbilt University Medical Center/Vanderbilt‐Ingram Cancer Center Nashville TN USA; ^3^ Department of Pathology, Microbiology and Immunology Vanderbilt University Medical Center Nashville TN USA

**Keywords:** chemotherapy, colorectal cancer, metastasis, passenger mutations, whole exome sequencing

## Abstract

Metastatic colorectal cancer (mCRC), particularly microsatellite stable (MSS) cases, often exhibits limited responsiveness to immunotherapy, leaving chemotherapy as the primary treatment option. While chemotherapy effectively targets tumor cells, its impact on the broader mutational landscape, including passenger mutations in large genes such as Titin (TTN), remains poorly understood. Passenger mutations, traditionally deemed biologically inert, may reflect tumor mutational burden (TMB) and influence treatment outcomes. In our study involving whole exome sequencing of paired primary and metastatic tumor samples from 22 mCRC patients, recurrent driver mutations in APC, KRAS, and TP53 were consistently observed. However, passenger mutations in large genes, particularly TTN, were notably enriched in chemonaïve specimens and associated with higher TMB. Chemotherapy‐treated samples exhibited a significant reduction in these mutations, suggesting selective depletion of hypermutated subclones. Our findings demonstrate that chemotherapy may selectively reduce passenger mutations in mCRC, potentially influencing the persistence of hypermutated subclones. This highlights the potential role of passenger mutation patterns and TMB as biomarkers for treatment response and raises the hypothesis that they could help guide immunotherapy considerations for patients with MSS mCRC.

## Graphical abstract

Changes in passenger mutation load and predicted immunotherapy response after chemotherapy treatment. Tumor cells rich with passenger mutations have increased sensitivity to chemotherapy. Correlation of passenger mutations with neoantigen load suggests highly mutated clones promote a more effective response to immunotherapy, and therefore, first‐line chemotherapy may limit response to subsequent immunotherapy due to selective loss of highly mutated clones.

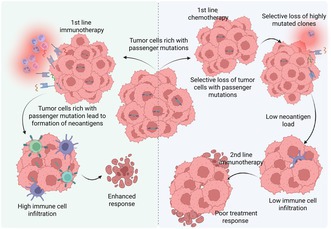

**Abbreviations**
AJCC: American Joint Committee on Cancer
CIN: chromosomal instability
COAD: colorectal adenocarcinoma
CRC: colorectal cancer
dbGAP: database of genotypes and phenotypes
DEL: deletions
FFPE: Formalin‐fixed paraffin embedded
FGA: fraction of genome altered
INS: insertions
mCRC: metastatic colorectal cancer
MSI‐H: high microsatellite instability
MSI‐L: low microsatellite instability
MSS: microsatellite‐stable
SNP: single nucleotide polymorphism
SNV: single nucleotide variant
TCGA: The Cancer Genome Atlas
TMB: tumor mutational burden
VANTAGE: VanderBilt Technologies for Advanced Genomics

## 1 Introduction

Colorectal cancer (CRC) is the second most common cause of cancer‐related mortality [1]. While early‐stage disease is often curable, metastatic colorectal cancer (mCRC) continues to carry a poor prognosis despite the availability of chemotherapy, targeted therapies, and, more recently, immunotherapy [2]. To improve patient outcomes, there is an urgent need to better understand the genomic alterations driving disease progression and therapeutic response in mCRC.

Molecular characterization of CRC has traditionally centered around driver mutations in genes such as KRAS, NRAS, BRAF, APC, and TP53, which regulate critical pathways like Wnt/β‐catenin and MAPK/ERK and directly influence clinical decision‐making [3]. APC mutations occur typically early in tumorigenesis, while TP53 mutations are associated with tumor progression and genomic instability [4]. KRAS, NRAS, and BRAF mutations are frequently mutually exclusive and play key roles in promoting proliferation and resistance to EGFR‐targeted therapies [5].

Microsatellite instability (MSI) status remains a cornerstone of CRC classification and treatment stratification. While MSI‐High (MSI‐H) tumors are well characterized and highly responsive to immune checkpoint inhibitors, MSI‐Low (MSI‐L) tumors occupy a less understood intermediate space between MSI‐H and microsatellite stable (MSS) phenotypes [6]. Emerging evidence suggests that MSI‐L tumors may represent a biologically distinct subset with subtle increases in mutation burden and genomic instability, though lacking the full hypermutated phenotype of MSI‐H tumors [7]. Whether MSI‐L status carries predictive or prognostic value remains a question of ongoing investigation.

While the driver mutational profile is well characterized for CRC and its metastases, the role of passenger mutations is less understood. Passenger mutations are random mutations that occur in cancer cells but do not play any direct role in tumor cell growth or survival [8]. These random mutations occur in the same cells as driver mutations and contribute to genomic instability and high mutational burden in tumors [9]. Although no causal relationship has been developed between passenger mutations and cancer progression, one study showed that increasing passenger load in cancer cells reduces the fitness of such cells and results in decreased progression of both primary and metastatic disease. [10] However, to date, little is known about the role of passenger mutations in therapeutic response in cancers especially those with poor prognosis, such as MSS CRC.

In this study, we present a comprehensive analysis of paired primary and mCRC samples using whole‐exome sequencing. We examine the frequency, distribution, and potential clinical relevance of passenger mutations in large genomic loci in MSS mCRC and explore how prior treatment with chemotherapy influences the mutational landscape.

## 2 Methods

### 2.1 Tissue collection and sample preparation

The study protocol adhered to the principles outlined in the Declaration of Helsinki for research involving human subjects. All formalin‐fixed paraffin‐embedded (FFPE) tissue (primary tumor, metastatic tumor, and normal adjacent tissue) and corresponding clinical data were collected between 2013 and 2018 with written consent of each subject at Vanderbilt University Medical Center. Study protocols were approved by the Vanderbilt Institution Review Board (IRB #010680 and IRB #101531). Appropriate IRB‐approved investigators determined study inclusion, and this information was de‐identified and HIPAA‐compliant prior to the release of data to key study personnel.

All FFPE blocks used for analysis were stored at room temperature until sectioned for DNA extraction. The first 10 μm of the tissue was discarded, then 5‐μm‐thick tissue sections were sectioned and mounted on uncharged glass slides. Separate tissue sections from the top and bottom of the serial sections used for DNA extraction were stained with H&E for quality control from each tissue block. H&E slides were reviewed for tumor percentage, areas containing the highest percentages of tumor were annotated, and then, the manual dissection of unstained sections was performed. DNA was purified using the QIAamp DNA FFPE tissue kit by Qiagen according to the manufacturer's instructions. A minimum of 3 sections per sample was required.

### 2.2 Whole‐exome sequencing

DNA samples were submitted in a single batch to Vanderbilt Technologies for Advanced Genomics (VANTAGE) for quality control analysis and whole‐exome sequencing. If quality was sufficient, DNA was sheared via Covaris, and libraries were prepared with the Illumina TruSeq Exome (45 Mb) panel and captured as a 9‐plex. Sequencing was performed on the Illumina HiSeq 3000 at Paired‐End 75 bp targeting ~50 m reads/sample (~100× coverage).

Preprocessing of sequenced reads was performed using nextflow v23.04.2 with the nf‐core/sarek [11, 12] v3.2.3 pipeline using the GATK GRCh38 genome assembly downloaded from the iGenomes data portal. Briefly, assessment of read quality and trimming was performed using fastp [13] v0.23.4, and then, reads were mapped using BWA‐mem v0.7.17. Strelka2 [14] was used for calling germline and somatic variants and variants were further annotated using SnpEff [15]. CNVkit [16] was utilized to identify copy number alterations, and MSIsensor‐pro [17] was used to score microsatellite stability. To calculate the fraction of genome altered (FGA), segments with a log_2_ fold‐change of at least 0.25 were considered as ‘altered’. Then, the size of all altered segments is summed and divided by the total genome size. Chromosomal instability (CIN) composite scores were calculated for each specimen to consider the extent of alterations using the FGA, the magnitude of alterations using the size‐weighted, absolute log_2_ fold change ratios for gains and losses, and the complexity of alterations from the total number of segment transitions per chromosome. Downstream analysis and visualization were performed in R v4.4.0 using maftools [18] v2.24.0.

For reanalysis of publicly available WES data from The Cancer Genome Atlas (TCGA) colorectal adenocarcinoma (COAD) dataset [19], we used cBioPortal [20, 21, 22]. Of the 594 samples, 206 were identified as treated (postchemotherapy).

### 2.3 Statistical analysis of paired treatment samples

For patients in which longitudinal specimens included at least one chemonaïve and one treated specimen (14/22 cases), we utilized a linear mixed‐effects model using the lme4 v1.1–37 R package. Treatment status was modeled as a fixed effect, and the patient ID was modeled as a random effect to control for patient‐to‐patient variation in baseline mutation rates. Statistical significance was evaluated at α = 0.05.

## 3 Results

### 3.1 Clinicopathology characteristics of mCRC cohort

Normal, primary colorectal, and paired specimens from various metastatic sites were acquired for 22 mCRC patients. The samples from metastatic sites were further categorized into regional (lymph nodes; *n* = 10) and distant, including peritoneum (*n* = 6), ovary (*n* = 8), small bowel (*n* = 4), spleen (*n* = 1), liver (*n* = 9), and lungs (*n* = 6). The known clinicopathological characteristics are summarized in Table 1. The majority of patients (59.1%) were over the age of 50, with a nearly equal distribution of males (45.5%) and females (54.5%). Notably, 77.3% of patients reported no family history of colorectal cancer. On histological evaluation, most tumors (77.3%) were moderately differentiated adenocarcinomas, while two patients had well‐differentiated and mucinous adenocarcinomas, and one patient had a poorly differentiated tumor. Most patients had their primary tumor located in the right colon (59.1%). In comparison, 8 (36.4%) patients presented with a left‐sided primary tumor, while only 1 patient had a tumor originating from the rectum. According to the American Joint Committee on Cancer (AJCC) staging, 19 (86.4%) of patients presented with stage III or IV disease and 3 (13.6%) with stage II disease. Additionally, 8 (36.4%) of the patients presented with synchronous metastasis and 11 (50%) of the patients presented with metachronous metastasis. All patients (95.5%) were classified as MSS except for one (ER5) which was MSI‐H. One patient (ER2) had three total specimens, two of which were pretreatment and were classified as MSI‐L (2.8% and 8.7% microsatellite stability sites mutated). However, the third specimen collected post‐treatment, had a reduction in mutated microsatellite sites (0.8%) and was classified as MSS.

**Table 1:** Clinicopathological characteristics of cohort with metastatic colorectal cancer (*n* = 22).

Patient characteristics (*N* = 22)		
Variable	Category	% (*n*)
Age	<40	13.6% (3)
	40–50	27.3% (6)
	>50	59.1% (13)
Gender	Male	45.5% (10)
	Female	54.5% (12)
Race	White	90.9% (20)
	Black or African American	4.5% (1)
	Unknown or unreported	4.5% (1)
Family history	Positive	22.7% (5)
	Negative	77.3% (17)
Smoking history	Positive	45.5% (10)
	Negative	54.5% (12)
Histology	Poorly differentiated	4.6% (1)
	Moderately differentiated	77.3% (17)
	Well differentiated	9.1% (2)
	Mucinous	9.1% (2)
Primary tumor site	Right colon	59.1% (13)
	Left colon	36.4% (8)
	Rectum	4.5% (1)
AJCC staging	Stage I	0.0% (0)
	Stage II	13.6% (3)
	Stage III	45.5% (10)
	Stage IV	40.9% (9)
MSS status	MSS	95.5% (21)
	MSI‐H	4.5% (1)

### 3.2 Mutational landscape of mCRC

**Fig. 1:**
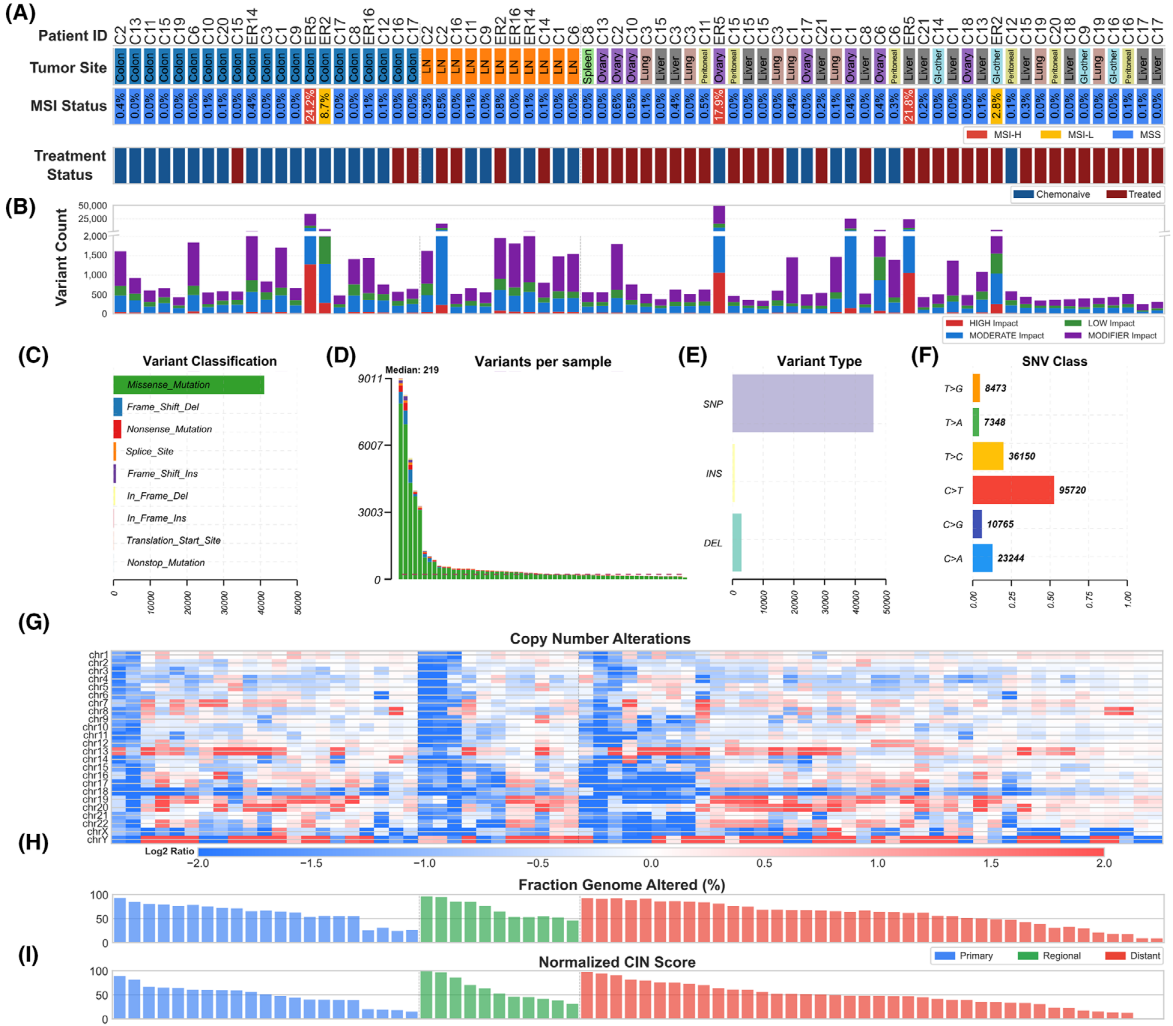
Genomic characterization of mCRC specimens. (A) Tumor site, microsatellite instability (MSI) status, and treatment status for each specimen. (B) Total variant count per specimen, stratified by predicted variant impact (high, moderate, low, modifier). (C) Distribution of variant classifications across the cohort, including missense, nonsense, frameshift, splice site, and in‐frame mutations. (D) Number of variants per specimen, with samples ranked in descending order and the median indicated. (E) Distribution of variant types categorized as single nucleotide polymorphisms (SNPs), insertions (INS), and deletions (DEL). (F) Frequency of single nucleotide variant (SNV) classes across the cohort. (G) Heatmap of copy number alterations across specimens. Columns correspond to specimens and rows correspond to chromosomes. Colors indicate log fold change for copy number gains (red) and losses (blue). (H) Fraction of genome altered and (i) chromosomal instability (CIN) score (normalized to 1) in each specimen, grouped by tumor sampling site (primary, regional, distant).

We first assessed the overall mutational architecture based on whole‐exome sequencing for the 72 samples. Each tumor specimen was annotated by anatomical location, treatment status, and overall microsatellite stability status, where mutations in more than 10% of microsatellite sites were considered MSI‐H, between 1% and 10% as MSI‐L, and fewer than 1% as MSS (Fig. 1A). About 93% of specimens were identified to be MSS, consistent with previous reports for mCRC [23]. Somatic variants were annotated by predicted impact and classified by the type of mutation (Fig. 1B,C). In general, the total number of somatic variants was consistent with microsatellite stability scoring, where cases with higher numbers of unique variants also had a higher proportion of mutated microsatellite sites. A median of 219 variants per sample was detected, with a maximum of 9011 variants, and single nucleotide polymorphisms (SNPs) were the most abundant variants identified (Fig. 1D,E). Consistent with larger cohort studies, the most abundant class of single nucleotide variants (SNVs) were cytosine to thymidine (C>T) mutations (Fig. 1F) [24].

The majority of mCRC display chromosomal instability (CIN), often characterized by widespread chromosomal abnormalities and correlated with mutations in common driver genes, such as APC, KRAS, and TP53 [25]. We observed a large number of copy number alterations across our specimens and calculated the fraction of genome altered (FGA) for each specimen (Fig. 1G,H). A CIN score was calculated using the FGA and the average magnitude of copy number changes weighted by the size of the genomic region containing copy number alterations, which was then normalized across specimens (Fig. 1I). Overall, most specimens display high CIN, but the FGA and normalized CIN scores were not correlated with the total number of variants or microsatellite stability status in our cohort.

### 3.3 Chemotherapy contributes to patterning of common mutations in mCRC

**Fig. 2:**
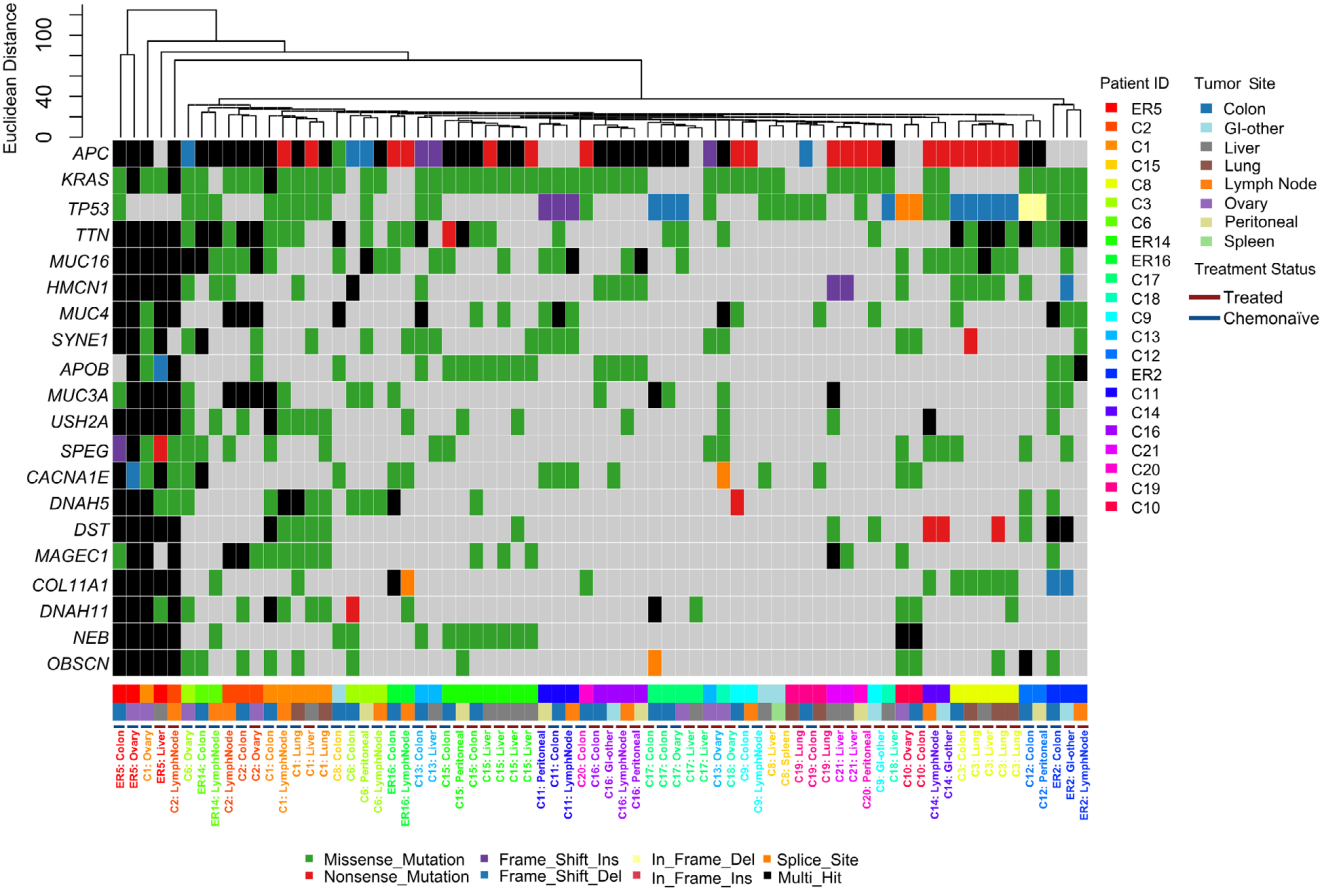
Mutational landscape and clustering analysis of mCRC samples. Rows indicate the top 20 most frequently mutated genes across the cohort and columns indicate unique specimens colored by patient identifier. Columns are further annotated with colored bars indicating specimen tumor site and treatment status at time of collection. Columns are ordered based on Euclidean distance of captured mutations, where specimens of similar mutational patterning are closer together. Cells on the oncoplot are colored by type of mutation.

We initially hypothesized that different sites of metastases would display different mutational patterns or enrichment for specific mutations. To assess this possibility, we performed hierarchical clustering for high‐ and moderate‐impact somatic variants and plotted the 20 most common mutations across our cohort (Fig. 2). The most frequently mutated genes were *APC* (79%), *KRAS* (74%), *TP53* (57%), and *TTN* (53%), in line with previously reported large cohort studies for primary and mCRC [26, 27, 28, 29, 30, 31, 32]. *KRAS* mutations were primarily missense variants, consistent with their role in gain‐of‐function oncogenic signaling [33]. In contrast, *APC* mutations were largely truncating events—nonsense, frameshift deletions, or multi‐hit mutations—typical of its tumor suppressor [34]. *TP53* showed a mix of missense and truncating mutations. Mutations in *SPEG*, though less common, were all truncating in nature and may implicate the gene in intracellular signaling dysregulation [35]. Other frequently mutated genes included *SYNE1* (31%), *USH2A* (28%), and *NEB* (25%), all of which have been associated with increased tumor mutation burden (TMB) and worse prognosis in prior studies [36, 37, 38].

By hierarchical clustering, paired specimens from the same patient, regardless of anatomical location or longitudinal collection time, displayed the highest similarity. To further determine if differences between metastases and primary tumors could be detected in our dataset, we performed clinical enrichment testing. However, no statistically significant mutations were associated with regional or distant metastases, or for any specific anatomical site compared to primary tumors. Interestingly, we observed that specimens collected after chemotherapy treatment tended to diverge from chemonaïve paired specimens, suggesting that chemotherapy has a selective effect on overall mutational architecture.

### 3.4 Chemotherapy selects against passenger mutations in mCRC

**Fig. 3:**
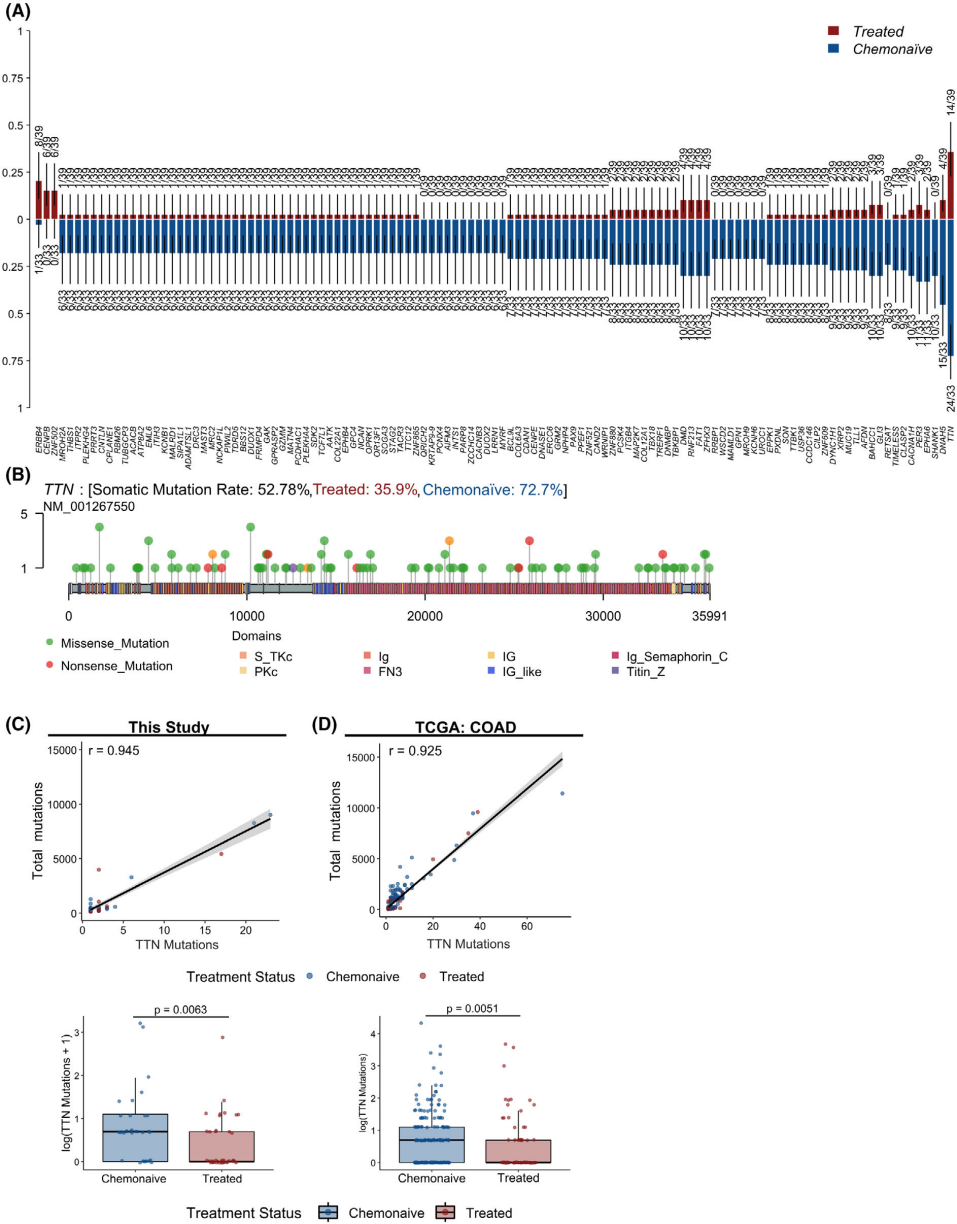
TTN mutation frequency and association with tumor mutation burden. (A) Histogram of enriched of genes with single nucleotide variants (SNVs) in colorectal cancer samples, stratified by treated (red) or chemonaïve (blue). (B) Lollipop plot of somatic mutations in the *Titin* (*TTN*) gene, showing the location and frequency of mutations across protein domains. Correlation between *TTN* mutation count and total tumor mutation burden (top) and box‐and‐whisker plots comparing *TTN* mutations in chemonaïve to treated samples (bottom) for this study (C) and from The Cancer Genome Atlas (TCGA) colorectal adenocarcinoma (COAD) dataset (D). Each point indicates a unique specimen, colored by treatment status. For correlations, Pearson's r is reported. For box‐and‐whisker plots, the median value for each group is marked by a thick black line and the resulting p‐value from a Wilcoxon‐ranked sum test is reported for each dataset. For this study, where pre‐ and postchemotherapy status is known, a pseudocount of 1 is added prior to the log of *TTN* mutations to visualize specimens that no longer displayed *TTN* mutations after treatment.

To determine which mutations were affected by prior chemotherapy treatment, we again performed clinical enrichment testing and identified 72 genes with differential mutation patterns between treated and chemonaïve specimens (Fig. 3A). To our surprise, the majority of mutations (114/117) were enriched in the chemonaïve group and mutations in only *ERBB4*, *CENPB*, and *ZNF502* were enriched in the treated group. The most common *ERBB4* mutation in our dataset, C1177T (R393W), was previously identified as an activating recurrent driver mutation in the ligand binding domain in colorectal, gastric, and melanoma cancers [39, 40, 41]. For *CENPB*, both mutations were identified in regions associated with binding of DNA in CENP‐B box sequences. Although the functional effects of these *CENPB* mutations are unknown, one mutation was a single base deletion that results in a frameshift and therefore may cause loss of CENP‐B function. In breast cancer, lower expression of CENPB was associated with decreased recurrence‐free survival [42], suggesting that mutations in *CENPB* may play a role in chemotherapy resistance. Similarly, *ZNF502* was identified as one of the two downregulated genes in multiple oxaliplatin‐resistant CRC cell lines, although the functional role of the mutations we observed is also unknown [43].

In contrast, one of the most commonly mutated genes in CRC, *TTN*, was highly enriched in the chemonaïve specimens (22 of 30 specimens). In our dataset, *TTN* was the fourth most commonly mutated gene (53% of specimens). We also observed that numerous other enriched genes mutated in the chemonaïve group encoded very large proteins. Therefore, we postulated that the frequency of passenger mutations, mutations that accumulate alongside driver mutations and that are not expected to directly contribute to tumorigenesis, was specifically enriched in the chemonaïve group. We plotted the variants in *TTN* and observed a random distribution of mutations, supporting that these variants are passenger mutations that occur with high frequency due to the large size of the gene (Fig. 3B).

To better understand how treatment alters the presence of *TTN* variants, we compared the correlated the total number of *TTN* variants in each specimen to the total number of variants and observed a significant positive correlation (Fig. 3C). Across all specimens, total *TTN* mutations were also significantly decreased post‐treatment. Furthermore, we utilized a generalized linear mixed‐effects model to explore the effect of chemotherapy on *TTN* mutations in cases that had both pre‐ and post‐treatment specimens available. This approach identified a statistically significant 40% reduction in *TTN* mutations post‐treatment (*P* = 0.0212). To further examine how treatment may alter the presence of *TTN* variants in a larger cohort, we accessed The Cancer Genome Atlas (TCGA) colorectal adenocarcinoma (COAD) dataset and classified cases based on their treatment history as chemonaïve or treated. Consistent with our observations, *TTN* mutations were highly correlated with overall mutational burden and chemonaïve samples demonstrated a higher number of *TTN* mutations compared with treated samples (Fig. 3D).

**Fig. 4:**
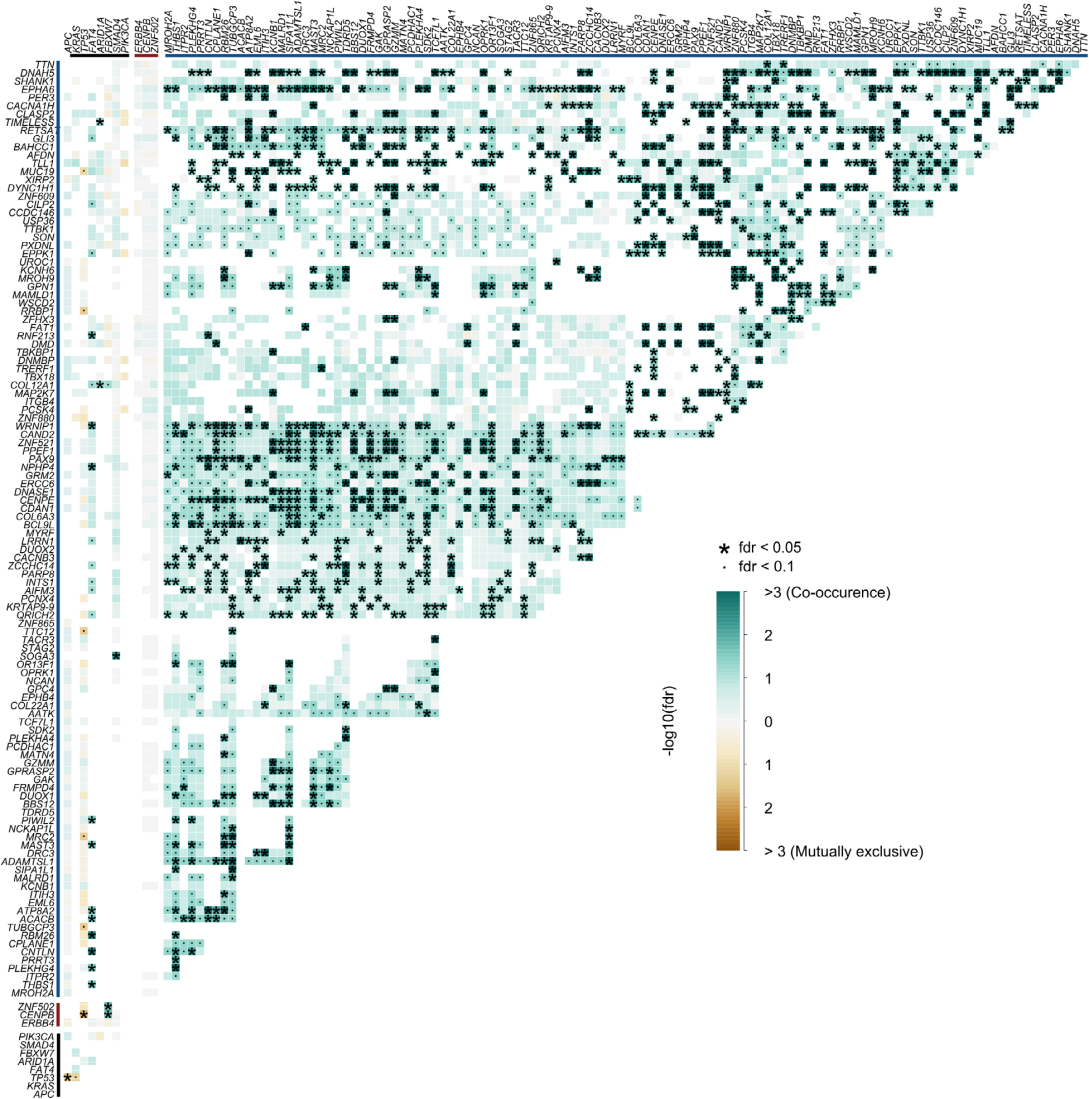
Pairwise co‐occurrence and mutual exclusivity of somatic mutations in mCRC samples. Co‐occurrence between selected colorectal‐associated driver mutations (black), treatment‐enriched mutation (red), and chemonaïve‐enriched mutations (blue; chemonaïve). Color indicates likelihood of co‐occurrence between mutations where high co‐occurrence is teal and low co‐occurrence is brown. Significance indicated by *P < 0.05.

Next, we performed a Pearson correlation between selected common driver mutations and mutations associated with treatment (Fig. 4). *ERBB4, CENPB, and ZNF502* were not significantly associated with any other mutations, and very few significant associations were observed for the common driver mutations. However, most of the genes enriched in the chemonaïve group were significantly co‐occurring. Taken together, these data highlight that passenger mutations in large genomic loci reflect the overall TMB and suggest that clonal tumor populations with high rates of passenger mutations may be most sensitive to chemotherapy.

## 4 Discussion

This study provides a comprehensive analysis of the mutational landscape in a cohort of 22 mCRC patients with matched metastases (72 samples), emphasizing the prevalence and potential clinical relevance of passenger mutations. As expected, *APC*, *KRAS*, and *TP53* mutations were the most common mutations [4]. In our cohort, only 4 specimens were identified with *BRAF* mutations, which are notably more common for MSI‐H cases [44].

*TTN*, is the largest gene body and encodes for the protein TITIN, known to play a significant role in muscle diseases and cardiomyopathies [45, 46]. Although mutations in this genomic locus have been associated with numerous cancers, mutations in *TTN* are largely predicted to have low phenotypic impact [47, 48]. While frequent mutations in large genomic loci, such as for *TTN*, have historically been regarded as passenger mutations and not relevant to clinical progression, recent evidence from others also suggests that these mutations may serve as a proxy for high TMB, particularly in tumors lacking MSI‐H status [49, 50]. In our cohort and in other large cohort studies, *TTN* mutations were frequent—often present in more than 40% of cases—and correlate strongly with total variant burden [51, 52].

Interestingly, we observed a lower prevalence of *TTN* mutations in chemotherapy‐treated specimens compared to chemonaïve ones, suggesting potential therapeutic selection against clones with high passenger mutation rates and/or *TTN*‐mutant clones. This finding raises the hypothesis that cells with high passenger mutation rates may be more immunogenic or vulnerable to DNA‐damaging agents. A study by Liu *et al*. identified that the presence of both *TTN* and *OBSCN* mutations predicted an immune‐hot subset that may respond better to immunotherapy [50]. We observed mutations in both genes to be reduced postchemotherapy in our cohort, warranting further investigation into cells with high passenger mutation rates and their response to immunotherapy or combination regimens. Indeed, the objective response rates in patients with MSS CRC who have received multiple lines of prior chemotherapy with monotherapy or dual immune checkpoint inhibitors range from 0 to 1.2% compared to 27% in chemonaïve MSS CRC [53, 54, 55, 56]. Multiple other studies have also shown that *TTN* mutants are predictive of chemotherapy response and that mutations in large genes can result in the development of neoantigens that drive functional endogenous immune responses in cancers such as lung and ovarian [57, 58].

Importantly, this study was performed on archival tissue from paired primary tumors and various metastatic sites and, in line with the expected rate, most cases were MSS. In this cohort, we were not able to detect significant mutations enriched in different sites of metastases. Other recent studies with larger sample sizes have attempted to identify metastasis‐associated variants and clonal progression with some success [26, 59, 60]. For this study, we chose to utilize whole‐exome sequencing to detect somatic variants. However, future studies leveraging whole‐genome sequencing would provide much more context for the mutational landscape and for clonality analysis. Large cohort studies often utilize curated mutation panels, such as the MSK‐IMPACT panel, and these approaches have greatly enhanced our understanding of disease progression in CRC [61]. Although not all large genes are included in the MSK‐IMPACT panel, *TTN* is included and would enable future studies to assess how chemotherapy or immunotherapy responses are altered in patients using *TTN* variants as a surrogate for passenger mutation abundance.

## 5 Conclusions

Taken together, these data highlight that passenger mutations may serve as a surrogate marker of overall TMB and that chemotherapy could influence the persistence of highly mutated clones. While preliminary, these findings support a growing body of evidence that the order of treatment may have a significant impact on the effectiveness of immunotherapy [62]. Currently, immunotherapy is primarily used in MSI‐H cases and in chemotherapy‐refractory cases [63, 64]. However, a shift towards first‐line immunotherapy has gained traction due to increased efficacy, which we speculate may be at least partially due to the availability of passenger mutation‐driven neoantigens that promote an effective immune response [65]. Although more comprehensive studies are necessary, we show that high‐frequency passenger mutations also occur in MSS cases and therefore hypothesize that certain MSS cases may have a better response to first‐line immunotherapy.

## 6 Limitations

Our study has several limitations. First, the relatively small sample size may reduce the statistical power and limit our ability to detect less frequent genomic alterations when comparing pre‐ and post‐treatment samples, including matched samples. Second, as this was a single‐center study, the findings may not be generalizable to broader patient populations. Larger, multi‐center follow‐up studies are necessary to further validate our observations and to assess how overall TMB or presence of *TTN* mutations can be used as biomarkers prior to treatment. Finally, functional preclinical experiments are required to understand the mechanisms underlying the observed reduction in passenger mutations following chemotherapy treatment and to determine how the loss of highly mutated clones corresponds to neoantigen presence and immunotherapy response.

## Conflict of interest

MTS, MAC, MBM, KBL, KW, and KI report that they have no conflicts of interest related to this work. KKC reports that they have consulted or been on the advisory board for Pfizer, Incyte, Exelixis, Bayer, ALX, Tempus, Taho, Agenus, Merck, Beigene, and Summit Therapeutics and have received research grants to the institution from BMS, Array, Incyte, Nucana, Merck, Pfizer, Calithera, Genentech, Seagen, Syndan, and Biomea.

## Author contributions

MTS, MAC, and KI conceived and designed the project. MTS, MAC, KBL, and MKW acquired the data. MTS and MAC analyzed and interpreted the data and created the visualizations. MTS, MAC, and KI wrote the paper. MBM and KKC provided data curation support. KKC and MKW provided resources. KI supervised the project, administered the project, and acquired funding.

## Data Availability

All raw and processed next‐generation sequencing data will be available upon reasonable request to the corresponding author.
